# Buffalo Academy of Medicine

**Published:** 1900-11

**Authors:** George F. Cott

**Affiliations:** Secretary


### Society Proceedings.

### BUFFALO ACADEMY OF MEDICINE.

*Section on Ophthalmology, Otology, Rhinology and Laryngology, September 17, 1900.*

Reported by GEORGE F. COTT, M. D., Secretary.

DR. F. WHITEHILL HINKEL, the chairman elect, opened the meeting with a few well-chosen remarks thanking the section for the honor conferred.

The chairman called attention to the necessity of having a separate set of by-laws to govern this section.

Upon motion of Dr. A. A. Hubbell, the following order of business was adopted: Reading of minutes; presentation of cases and exhibition of specimens; reports of committees; unfinished business; new business (including nomination and election of officers); reading of papers and discussion; adjournment.

Motion of Dr. A. A. Hubbell—Nomination and election of officers to take place at regular meeting in month of May each year and a notice thereof be sent out with regular postal card notice. Carried.

Dr. G. F. Cott presented a larynx which he had removed from a woman 63 years of age, suffering considerable pain, especially during the act of swallowing; had coughed for two years, but did not notice any serious symptoms until about six months ago. About that time Dr. Hebenstreit saw her and prescribed for her. From the general appearance he thought she had carcinoma of the larynx. In March of this year Dr. Hintz was called in and he in turn consulted with Dr. King, who also made a diagnosis of carcinoma. Dr. King asked me to examine the woman and I found her as above described. The larynx was covered with a yellowish exudate, anterior surface infiltrated and left arytenoid very much swollen, large glandular enlargement on left side of neck. April 11, 1900, the larynx was removed at Riverside Hospital, with the assistance of Drs. Jacob Meyer, King and Thompson. The larynx was removed in twenty-eight minutes. The esophagus was stitched to the skin laterally and the trachea to the skin anteriorly to prevent exudate from running down into the bronchi. Wound was then packed. Patient did apparently well, but died suddenly two hours later.

Dr. F. W. Hinkel had observed five or six cases which were operated upon, the first one was a brilliant success, the rest died within a short time. His experience taught him never to urge operation for carcinoma of larynx.

Dr. Hinkel suggested that proper apparatus for examination of patients be procured. Dr. Bennett said that the council had given section permission to get whatever they needed.

Dr. A. L. Benedict read a paper entitled,

### EYE STRAIN OR GASTRIC REFLEX—WHICH?

The writer, while admitting the possibility of vomiting, headache, and the like, as a result of eye-strain, considered that dyspeptic trouble of any duration, characterised by these symptoms, was apt to be of independent origin. He believed that the oculists of Buffalo shared this view as so many patients had been referred to him for treatment of digestive conditions, which might have been retained by the oculists if they held the ideas of eye-strain, sometimes attributed to this specialty. A number of cases were cited, mainly of gastric subacidity, but including hyperchlorhydria, gastric catarrh, due to hepatic sclerosis, gastric dilatation, gastroptosis, with movable right kidney, etc. As is the experience of specialists in gynecology and, probably other specialties, he had not been able to connect definite symptoms with definite gastric conditions, except for certain minor details.

Dropping the term eye-strain in its usual sense and using it merely to signify overwork of the eyes, whether normal or subject to refractive errors, he believed that the nausea and vomiting of scorchers was due largely to eye-strain, on account of the effort to focus on the road while subject to the vibration of the bicycle, and also on account of the rapid changes of illumination as from the sun shining through the pickets of a fence or through the waving branches of trees. Persons using their eyes excessively in viewing landscapes from trolleys or steam cars, in watching theatrical performances, or in any other way, were liable to gastric reflex. Sick headache, generally characterised by failure of hydrochloric acid and organic acidity, instead of by hyperchlorhydria, as often thought, was frequently due to eye-strain in the same sense, though not invariably as has been declared.

Discussing the physiology of gastric symptoms from eye-strain, Dr. Benedict called attention to the fact that special sense excitations as such, did not cause nausea unless the sense of sight or of equilibrium was affected. Even nausea from irritation of taste and smell was essentially psychic, as shown by persons eating with relish and without nausea such substances as high meat, Limburger cheese, onions, and the like. The control of equilibrium was largely due to sight and these two senses were closely connected in the corpora quadrigemina and geniculata. Dr. Benedict was somewhat sceptic as to the supposed function of the semicircular canals and believed that many ideas of equilibrium were due to pneumogastric endings in the liver and possibly in the stomach. The inertia of the liver adapted it to this purpose and elevator sickness, nausea from swinging in hammocks, etc., showed the susceptibility of the system to nausea from irregular movements of the liver. On the other hand, quite violent and irregular movements of the head alone, do not usually produce nausea.

In reply to a question as to statistics, the writer said that he had abandoned the attempt to prepare them, as they would amount practically to saying whether patients did or did not wear spectacles. As so few eyes were perfect, the demonstration of refractive error was not a satisfactory explanation of nausea, headache, etc., unless the patient recovered rapidly from the latter after using glasses, and unless the absence of organic digestive trouble was demonstrated physically. However, he made it a practice to refer patients for examination of the eyes, unless they had been recently refracted.

DISCUSSION.

Dr. C. G. Stockton felt some hesitation in opening the discussion since he did not grasp the conclusion reached by Dr. Benedict. It is no doubt very valuable, but he did not quite understand the paper as to the conclusion. Dr. Benedict did not go quite far enough as to therapeutic values regarding eye-strain. In the beginning of my career I had many disappointments in treating eye-strain, hence have modified very much my conclusions as to causation, relief, and the like. I profited first by suggestions of Dr. Hinkel, who called my attention to the matter. But things are more clear to me now, and I firmly believe that eyestrain is often the cause of disturbances of the stomach. There is, however, no one thing the cause, but a number of them together; four or five might do no harm, but add the sixth and you find the disturbance to occur. Refractive errors cause stomach and intestinal trouble from astigmatism, ametropia, and muscular errors. People suffering from such troubles have also other causes like over-work, lessened oxygenation, worry, sexual life, etc. Four factors may come together without causing an explosion, then adding the fifth and the mischief is done; eye-strain is one of those factors, and patients get better when eye-strain is relieved, but that is generally not enough to relieve all the symptoms; the others must receive separate attention. The eyes must be watched, of course. Some affections as those of railroad men, hysterical subjects, and other causative factors are sometimes hard to relieve, often even impossible. I consider it an important matter to have eyes corrected when there is stomach trouble.

Dr. A. G. Bennett—The ophthalmologist is hardly in position to discuss this question. He sees his patient usually but a short time and then refers him again to his physician. Many cases of eye-strain, however, have been relieved by the ophthalmologist's efforts alone of which I have ample evidence. Of course, all cases are not due to eye-strain. The most brilliant success was a case of hypophorea.

Dr. A. A. Hubbell—Dr. Benedict seems to mean that reflex stomach trouble is not due to eye-strain at all. His definition of eye-strain I take exception to. He says eye-strain is over-work. It is true we understand it to be overwork in another sense, to much work on muscles through some errors of refraction or motility, which leads to various reflexes. I take a medium ground on the subject which must be studied from all points and some one thing will bring to a climax. Vomiting may be caused by refractive error. The cases are very complex and must be clearly studied. On many points I agree with Dr. Stockton and Dr. Bennett. These patients generally go back to the general practitioner.

Dr. L. Burrows related a case of anisometropia, referred to him by a general practitioner; the peculiar condition of the stomach is generally associated with it.

Dr. F. W. Hinkel— From personal experience I know of relations existing between stomach and eye; the eyes are susceptible to irritation by the stomach. The stomach produces some form of toxemia causing difficulty in the use of the eyes.

Dr. A. A. Hubbell—Dr. Benedict is right in his statement of the myope. The ciliary muscles are not relaxed to the utmost and the stimulus of convergence and accommodation taken off. Most myopes are conscious of eye-strain.

Dr. J. J. Finerty—Dr. Hubbell, I would like to have you show me some cases. If eyes don't diverge there is no strain.

After some remarks by Dr. Blaauw, Dr. Benedict closed. He felt gratified at the free discussion.

Number of fellows present, 23.

---

### *Section on Obstetrics and Gynecology, September 25, 1900.*

Reported by WM. G. RING, M. D., Secretary.

THE regular monthly meeting of this section was held at the Academy rooms, Tuesday evening, September 25, 1900, at 8.30 p.m.

The meeting was called to order by the chairman, Dr. A. J. Colton, at 8.45 p.m.

The minutes of the previous meeting was read and approved.

Dr. C. E. Congdon read a paper on Obstetrics, Viewed From a Gynecological Standpoint. (See page 260.)

The paper was discussed by Dr. Grosvenor, who protested against the recommendation that vaginal irrigation should be practised before the birth of the child in obstetrical cases.

Dr. Wm. G. Taylor asked if repair of cervical lacerations was advisable immediately after labor, and suggested that the use of chloroform in the second stage of labor had in his practice prevented, to a large extent, lacerations of any variety.

Dr. STEPHEN Y. HOWELL said that the paper was full of meat and contained many valuable suggestions. He recommended close attention to cleanliness without regard to surroundings of patient. He thought that many obstetricians, even prominent ones, were at times very careless in regard to introduction of septic matter, and cited a case where he was called and found the physician dipping blood from the bed with an ordinary kitchen dipper; was greeted with a hearty hand-shake, and the same hand almost immediately was introduced uncleansed into the vagina of the patient. Germs are often carried into the vagina from unclean labia and mons. In lacerations the wounds are gaping for both venous and lymphatic infection and should be closed as soon as possible. Dr. Howell recommended the reading and publishing of such papers as that just read by the essayist.

Dr. C. E. Congdon, in closing, pointed out to Dr. Grosvenor that he had misunderstood him; that his views were the same as that of the paper; that he was very much pleased with the reception of his essay and its discussion; and stated that if only the physician had full charge and also the selection of the nurse, it would make a difference of 50 per cent. as regards results in obstetrics. Personally, the essayist had not been successful in preventing lacerations by use of chloroform. He agreed with Dr. Howell in his remarks.

Dr. FRANCIS E. FRONCZAK read a paper, entitled, A Few Glimpses, Mostly Medical, of Lands Across the Seas. (See page 255.) He related some of his experiences in the Azore Islands, Spain, Gibraltar, Africa, Italy and Austro-Hungary.

The paper was discussed by Drs. Congdon, Grosvenor and Krauss.

Dr. HENRY J. DOWD reported a case of vaginitis.

Number of members present, 26. Meeting adjourned at 9.30 p.m.

